# Physiology education for intensive care medicine residents: A 15-minute interactive peer-led flipped classroom session

**DOI:** 10.1371/journal.pone.0228257

**Published:** 2020-01-24

**Authors:** Bjoern Zante, Wolf E. Hautz, Joerg C. Schefold

**Affiliations:** 1 Department of Intensive Care Medicine, Inselspital, Bern University Hospital, University of Bern, Bern, Switzerland; 2 Department of Emergency Medicine, Inselspital, Bern University Hospital, University of Bern, Bern, Switzerland; University of Rome 'La Sapienza', ITALY

## Abstract

**Introduction:**

In acute care medicine, knowledge of the underlying (patho)-physiology is of paramount importance. This may be especially relevant in intensive care medicine, where individual competence and proficiency greatly depend on knowledge and understanding of critical care physiology. In settings with time constraints such as intensive care units (ICUs), time allotted to education is often limited. We evaluated whether introduction of a short, interactive, peer-led flipped classroom session is feasible and can provide ICU residents with a better understanding of critical care physiology.

**Materials and methods:**

Using the flipped classroom concept, we developed a 15-minute peer-led interactive “physiology education” session to introduce a total of 44 residents to critical care physiology. Using a nine-item electronic survey with open questions and a five-point Likert scale, we analysed the overall concept with regard to feasibility, motivation, and subjective learning of critical care physiology.

**Results:**

The overall rate of response to the survey was 70.5% (31/44). The residents reported that these sessions sparked their interest (p = 0.005, Chi square 10.52), and that discussion and interaction during these sessions had promoted their knowledge and understanding. Both novice and experienced residents reported that new knowledge was imparted (both p<0.0001, Chi-square 32.97 and 25.04, respectively).

**Conclusions:**

In an environment with time constraints such as the ICU, a 15-minute, interactive, peer-led flipped classroom teaching session was considered feasible and generally appeared useful for teaching critical care physiology to ICU residents. Responses to questions on questionnaires indicated that teaching sessions sparked interest and increased motivation. This approach may theoretically induce a modification in professional behaviour and promote self-directed learning. We therefore support the use of peer-led flipped classroom training sessions in the ICU. Whether these sessions result in improved ICU care should be addressed in subsequent studies.

## Introduction

In intensive care medicine, knowledge of underlying (patho)-physiology is of paramount importance. Further, an understanding of the physiological consequences of therapeutic interventions appears to be crucial, as proposed by Scribonius Largus around 47 AD in the principle “*primum non nocere”*, and Jean-Louis Vincent calling for “… *just good medicine*, *made on the basis of well-known physiology*” [1] in 2018. Thus, individual competence and proficiency in critical care may depend greatly on a doctor’s knowledge and understanding of critical care physiology.

Medical physiology is challenging to learn and teach [2–4]. Unfamiliarity with physiology may cause residents to struggle with learning new content [5], and limited prior knowledge of physiology may explain their difficulty to assimilate new knowledge [6]. The subsequent increased cognitive load of this new knowledge, combined with no or limited connection to pre-existing knowledge, may reduce the effectiveness of curricula or lectures [7, 8]. Additionally, many physiology topics may be regarded as specific to intensive care medicine [9, 10] and are often not included in curricula in medical schools [11]. When time is limited, education may also compete with clinical work and administrative responsibilities (e.g., documentation) [12]. In the ICU, clinical instructors are thus often confronted with the challenge of providing high-quality urgent medical treatment in addition to effective teaching. Novel approaches in the ICU thus require improved learning strategies [13, 14].

### Conceptual framework of peer teaching

Several different perspectives on peer teaching were identified: first, the cognitive and meta-cognitive level, and second, the affective motivational level of learning (student, teachers) [15]. Learning is defined as extension of existing knowledge, modification of cognitive schemes, and adjustment for adequacy and efficiency. Teaching aims to support all of these. It was postulated that a semantic network close to that of the learner (referred to as “*cognitive congruence*”) has a positive impact on understanding of needs and make learning more effective [16, 17].

Educational psychology demonstrates that learning is optimised if the distance between the already known/understood and the new learning content is selected well [18]. Peers may perceive this distance more accurately than experts, who may have misconceptions with regard to learners’ needs and may misunderstand learners’ cognitive difficulties in incorporating new information [19]. Moreover, peer-led teaching may motivate learning due to *social congruence*, which stimulates younger learners who are taught by their peers [20] and may be beneficial in fostering the learning of all peers [21]. The interactive setting and discussion may encourage residents to study particular topics in more detail and may help them develop more interest in the physiology-related content, especially in cases of diverse background knowledge [22].

In preparing to use a cognitive strategy, the peer serving as teacher determines his/her own goals and priorities. Based on the discovery theory of learning, this personal goal-setting is important for learning effects [23]. In the phase of presentation, verbalisation, recitation, and interaction with peers are known as important factors for learning [24]. Role theory explains how feelings and behaviour influence each other [25] and peer-teacher can foster their self-confidence. In addition, peer-teacher benefit from their own teaching [26].

### Conceptual framework of the flipped classroom

The flipped classroom concept reverses the traditional elements of a lecture. Learners are first introduced to the learning content and afterwards in the face-to-face time during the session they have the opportunity to apply this new knowledge and to engage in critical thinking. Based on the andragogy theory of learning, adult learning involves six principles: 1) the need to know and understand the benefits of learning, 2) the concept of individualised learning, 3) readiness to learn, 4) the need for applicable and relevant content, 5) pre-existing knowledge, and 6) motivation to learn [27]. Adult learners take responsibility for personal learning if they are motivated [28]. Hence, active learning as required in the flipped classroom concept may motivate the residents and fulfil their educational needs. Ideally, new knowledge is connected to pre-existing knowledge [29]. The constructivism theory defines the learner as the creator of his own knowledge based on pre-existing knowledge, and fosters this learning through social interaction [30]. Even in situations of dissonance, when a problem cannot be solved due to lack of prior knowledge, self-directed learning and reflective processes may help a learner to gain new knowledge and adapt existing knowledge [31]. Hence, the flipped classroom approach allows students to vary the amount of time needed for learning, determined by their needs and previous subject knowledge.

The aim of this investigation was to analyse a novel educational concept. We evaluated whether it is feasible to use a short, interactive, peer-led flipped classroom session to help ICU residents understand critical care physiology.

## Materials and methods

### Participants

Participants in the “physiology education” program were residents in a tertiary care academic hospital. Approximately 6,500 patients are treated per year in our multidisciplinary Department of Intensive Care Medicine, which comprises 37 intensive care and 20 intermediate care beds. Patients with all types of organ failures, multi-organ failure, including extracorporeal membrane oxygenation (ECMO) are treated.

The residents in our study came primarily from internal medicine (INT), neurology (NEU), neurosurgery (NCH), and visceral surgery (VS) and worked in our department for six month. Anaesthesiology (AN) residents typically serve for 6 to 12 months, and residents studying intensive care medicine (ICU) typically have more than 12 months of training in our department. Residents without specialisation in intensive care medicine were defined as novice residents, while residents who were being trained in intensive care medicine were defined as experienced residents. This variety provided an opportunity to foster learning in a peer-taught setting based on a corresponding framework. Over a period of 6 months, 44 residents participated in “physiology education” sessions and were invited to participate in a voluntary survey.

### Educational concepts: Learning objectives

Learning objectives of the “physiology education” programme embraced both basic physiology concepts and specific critical care physiology considered highly relevant for daily ICU work. Peer-reviewed articles from top-ranked medical journals provided an overview of (patho)-physiologic topics. (Key examples of a total of 50 topics are provided in Table 1).

**Table 1:** Examples of critical care physiology topics.

Hypoxaemia due to increased venous admixture–influence of cardiac output on oxygenation [32]
The role of venous return in critical illness and shock–part I. Physiology [33]
Bench-to-bedside review–carbon dioxide [34]
Serum chloride levels in critical illness–the hidden story [35]
Microcirculatory dysfunction in sepsis pathophysiology, clinical monitoring, and potential therapies [36]
Use of ScvO_2_ to guide therapy [37]
Bench-to-bedside review: an approach to hemodynamic monitoring–Guyton at the bedside [38]
Hemodynamic consequences of severe lactic acidosis in shock states–from bench to bedside [39]
Pulmonary capillary pressure [40]
Red blood cell rheology in sepsis [41]
Understanding wasted/ineffective efforts in mechanically ventilated COPD patients using the Campbell diagram [42]
Heart failure and kidney dysfunction: epidemiology, mechanisms and management [43]

### Educational concepts: Design and setting

**Fig 1:**
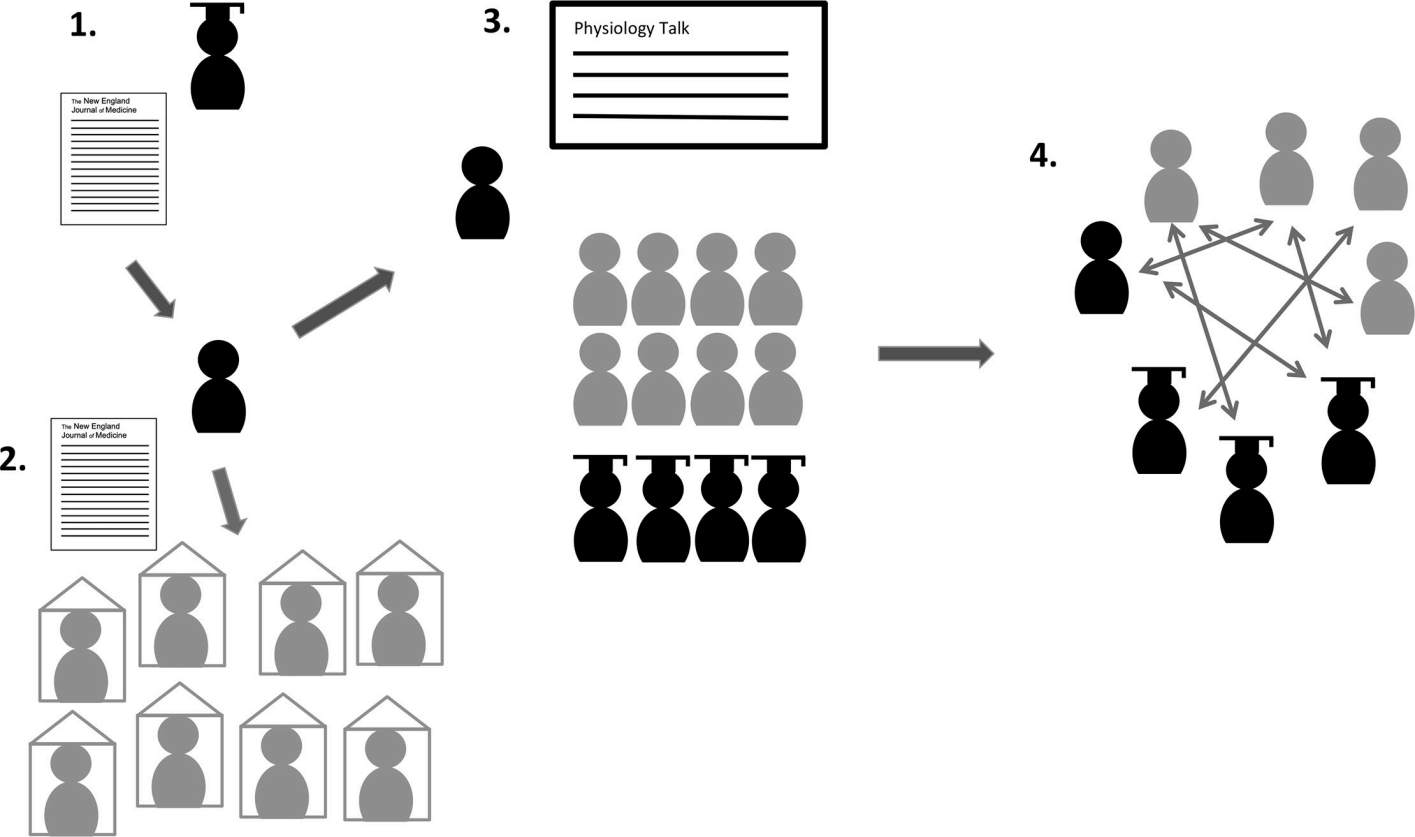
Setting of the peer-led flipped classroom concept. 1. Education coordinator sends article to peer-teacher, 2. Faculty members prepare learning material individually prior to the session. 3. Peer teaching during the session. 4. Interactive peer-led discussion is held to answer remaining questions, clarify concepts, and trigger critical thinking.

In the monthly departmental educational leaflet, bi-weekly “physiology education” was announced. The curriculum coordinator sent each week’s article to the pee-teacher.–All medical ICU staff (residents, registrars, consultants) received the article 5–7 days before teaching sessions, as based on the flipped classroom concept. According to this concept, faculty prepared learning material individually prior to the session. The peer-teacher prepared the session and was responsible for presentation of the article. During each session, time was used to discuss remaining questions, clarify concepts, and trigger critical thinking [44]. Attending consultants and registrars provided more detailed explanations triggered by the learners’ questions. This approach seemed useful to provide our “novice” residents with basic knowledge of physiology (Fig 1). Often, these novice residents were given a perspective extending beyond their own knowledge of physiology. More experienced residents had the opportunity to deepen their understanding of physiology in discussions, with explanations provided by expert attending consultants [29, 45]. Due to the time constraints in the ICU, a short and compact teaching session (15 minutes) was chosen.

### 9-item online survey

Participating ICU residents (observational period from March to December 2018) received an electronic link to a nine-item online survey. Participation was voluntary and anonymous. The survey dealt with the feasibility of the general concept, potential gains in knowledge, effectiveness, encouraging potential, and assessment of complexity. A 5-point Likert scale (1 = “totally agree”, 3 =“neutral” and 5 = “totally disagree”) (Table 2) was chosen. In addition, all participants could suggest changes. Background of the medical professionals and their amount of experience in intensive care medicine was noted.

**Table 2:** Survey questions using a 5-point Likert scale.

1. I have learned something new in the “Physiology education” session
2. I already knew something about the topics, but I learned something extra
3. I can apply the knowledge in daily practice
4. The complexity of the topics was appropriate
5. The “Physiology education” session has sparked my interest in the presented topics
6. The duration of the “Physiology education” session was appropriate
7. The discussion and interaction promoted my knowledge and understanding
8. The topics were presented at the right level
9. The “Physiology education” session is valuable

### Statistical analysis

A descriptive analysis was deliberately aimed for. Statistical analysis was performed using R Version 3.4.4 (R Foundation for Statistical Computing, Vienna, Austria). A one-way chi-square test proposing the null hypothesis of equal frequencies for positive (“totally agree” and “agree” categories) and neutral and negative (“Disagree” and “Totally disagree”) responses on the 5-point Likert scale was used. A p value <0.05 was considered significant.

## Results

Forty-four residents working in our multidisciplinary ICU were invited to participate in the electronic survey. The overall response rate was 70.5% (31/44). Residents’ primary medical background was anaesthesia (n = 5; 17.9%), internal medicine (n = 9; 28.6%) visceral surgery (n = 4; 14.3%), neurology (n = 1; 3.6%), ICU residents in training (n = 9; 28.6%), and other disciplines (n = 2; 7.1%). Data were missing for one participant. The amount of experience in the field of intensive care medicine was ≤6 months for 50% (n = 15), between 7 and 12 months for 13.3% (n = 4), and >12 months for 36.7% (n = 11), with missing data for one participant. Fourteen (45.2%) of the participating residents presented a physiology topic in the role of peer teacher in the “physiology education” programme.

### 9-item questionnaire results

Responses are given in Table 3 (plotted for distribution, frequency, percentage, median, p value and chi-square value). Questions 4, 6 and 8 referred to whether the teaching concept and topics were appropriate. In the analysis of question 4 (“The complexity of the topics was appropriate”), the distribution of responses on the Likert scale was significantly different. Analysis of question 6 (“The duration of the Physiology education session was appropriate”) and question 8 (“The topics were presented at the right level”) showed a significant difference in the distribution of responses on the Likert scale. Questions 1–3, 5 and 7 were intended to verify whether the “Physiology education” was encouraging to deal with ICU-physiology. For question one (“I have learned something new in the “Physiology education” session), question two (“I already knew something about the topics, but I learned something extra”), and question three (“I can apply the knowledge in daily practice”), distribution of responses on the Likert scale was significantly different. In novice residents, new knowledge was created (p<0.0001; Chi-square 32.97), and in experienced residents, additional knowledge was gained (p<0.0001, Chi-square value 25.04). In the analysis of responses to question five (“The “Physiology education” session has sparked my interest in the presented topics”), question seven (“The discussion and interaction promoted my knowledge and understanding”), and question nine (“The “Physiology education” session is valuable”), the distribution of responses on the Likert scale was significantly different.

**Table 3:** 9-item questionnaire results.

	Completely agree 1	Agree 2	Neutral 3	Disagree 4	Completely disagree 5					
	%	%	%	%	%	Responses N	Median	Interquartile range	p value	Chi square value
I have learned something new in the “Physiology education” session	25.81 (n = 8)	54.84 (n = 17)	19.35 (n = 6)	-	-	31	2.0	0.75	<0.0001	32.97
I already knew something about the topics, but I learned something extra	16.13 (n = 5)	58.06 (n = 18)	22.58 (n = 7)	3.23 (n = 1)	-	31	2.0	0.75	<0.0001	25.03
I can apply the knowledge in daily practice	6.45 (n = 2)	61.29 (n = 19)	16.13 (n = 5)	16.13 (n = 5)	-	31	2.0	1.0	0.0003	16.52
The complexity of the topics was appropriate	19.36 (n = 6)	35.48 (n = 11)	16.13 (n = 5)	22.58 (n = 7)	6.45 (n = 2)	31	2.0	2.0	0.03	7.23
The “Physiology education” session has sparked my interest in the presented topics	12.9 (n = 4)	38.71 (n = 12)	41.94 (n = 13)	6.45 (n = 2)	-	31	2.0	1.0	0.005	10.52
The duration of the “Physiology education” session was appropriate	19.36 (n = 6)	67.74 (n = 21)	9.68 (n = 3)	3.23 (n = 1)	-	31	2.0	0.0	<0.0001	40.52
The discussion and interaction promoted my knowledge and understanding	16.13 (n = 5)	51.61 (n = 16)	22.58 (n = 7)	9.68 (n = 3)	-	31	2.0	1.0	0.0002	17.29
The topics were presented at the right level	12.9 (n = 4)	48.39 (n = 15)	25.81 (n = 8)	12.9 (n = 4)	-	31	2.0	1.0	0.003	11.68
The “Physiology education” session is valuable	38.71 (n = 12)	35.48 (n = 11)	19.36 (n = 6)	6.45 (n = 2)	-	31	2.0	1.75	<0.0001	24.07
Average percentage	18.64	50.18	21.51	8.96	0.72					

Responses were given as frequencies and percentages, computing one-way chi-square test under the null hypothesis of equal frequencies for positive (“totally agree”, “agree”) and neutral and negative (“disagree”, “totally disagree”) responses on the 5-point Likert scale

### Open questionnaire questions

Seventeen residents (54.84%) responded to the open question “How can we improve the physiology education sessions?” (Table 4). They addressed five aspects. First, the frequency of possible participation was considered to be too low due to shift work. In general, offering more sessions was suggested. Second, for the interactive part involving more experienced consultants and registrars, it was noted that the level of discussion and interactive performance depended on which ICU consultants and registrars participated. Third, additional bedside teaching concepts were proposed to complement physiology sessions in a practical format. Further, problem-based learning and case-based learning were proposed. Fourth, several comments addressed the complexity of the topic. Some participants remarked that the participating residents started at different knowledge levels. Fifth, prolonging “physiology education” sessions was proposed.

**Table 4:** Sample responses to the question “How can we improve the physiology education sessions?”.

1. … better to make a short presentation by one of the consultants/registrars…”
2. “The session per se is a great thing! The problem is more that in 6 months I was able to participate only twice (due to shift work, compensation).”
3. “Topics are often too complex. It would be much easier if the residents were able to choose the topics by themselves and would be more practice-relevant in terms of skills training.”
4. “More discussion at the end with consultants and registrars. The session is enhanced by the presence of certain consultants!”
5. “More basics, then into the depth of the topic, because residents start at a different level”
6. “Do not use [published] papers for preparation, but rather basic physiology books or intensive care medical books. As problem-based learning. Take some more time, e.g. 30 min”
7. “Mostly the session consists of a presentation. Discussions would be desirable, but hardly occur. At the end of the presentation discuss an imaginary case …”
8. “Level may sometimes be a bit higher. Discussion should be sought more actively, with question slides at the end”
9. “more practical relevance”
10. “I think the session is good, and it is meaningful that the topics are selected according to the education-level of the lecturer”
11. “Session in the morning. Preferably right after the handover from the nightshift”

## Discussion

We observed that performing a 15-minute session aiming to teach critical care physiology was feasible in the ICU. Further, it appeared to have favourable effects on learning in ICU residents and may also have sparked interest in critical care physiology.

In the present survey, it appeared that the heterogeneity of participating ICU residents, given their different background education, made selection of suitable articles challenging. In particular, it seemed challenging to anticipate how much they already knew about the topics in an effort to prevent under- or overestimation of background knowledge [46, 47]. Responses to questions and some written comments addressed the appropriateness of topics and presentations, as well as revealing some evidence for a misconception of background knowledge in residents. However, the “physiology education” sessions overall were judged to be valuable, and residents reported that they learned new medical concepts. Importantly, in the questionnaire, learning effects were only measured by self-assessment, with all its inherent limitations [48]. On the other hand, in the subjective self-reported assessments, teaching sessions appeared to spark the interest of residents and likely increased intrinsic motivation. This motivation, enjoyment, and inherent satisfaction may be key to promoting intrinsic motivation in medical education [49]. Further, it may theoretically initialize and inspire further self-directed learning [50, 51] and increase performance [52, 53], even with short teaching sessions. Importantly, given the limited time available, it seemed crucial that the presentations were clear, meaningful, and enjoyable. Hence, interactive sessions seemed to enhance learners’ motivation as well as engagement–which was previously shown to increase performance [22, 54].

Some participants proposed more frequent sessions. Initially, we implemented sessions on a bi-weekly basis. After completion of the analysis presented here, a weekly session was chosen, as a relevant proportion of the residents could not participate in all sessions due to shift work.

Further, written comments suggested that interaction and discussion may have been influenced by participating consultants and registrars. Indeed, a teacher’s ability to teach, his/her interpersonal skills, and his/her personal/professional abilities may influence learners’ preference for that teacher [55]. Moreover, the subject-specific enthusiasm teachers convey may directly relate to learners’ performance [56]. Finally, the interaction between teachers themselves, as well as between learners, and the organisation may influence motivation [57].

The third aspect of the written comments was the proposal that sessions on physiology take place at the bedside, but a lack of suitable patients could make this difficult. Critical care patients usually have complex problems, with multiple instances of (patho-) physiological dysfunction. This could present residents with additional cognitive load [7, 8]. Case-based and problem-based learning were also proposed. However, despite the advantages of a patient-centred approach to learning physiology [58, 59], we decided on a teaching format that used a presentation focused on one specific topic to ensure a systematic curriculum independent of actual patients.

### Limitations

Our analysis has important limitations. In general, all limitations inherent to self-assessment must be considered when interpreting our findings [48]. However, self-assessment may be the only methodological approach useful in assessing whether participants subjectively judged the sessions useful, whether participants were satisfied with the learning content, and whether new information was acquired. Based on Kirkpatrick’s framework of training evaluation with this educational concept of a 15-minute “physiology education” session residents gained satisfaction (Level one of Kirkpatrick’s framework). However, reliable assessment of newly acquired knowledge (Level two of Kirkpatrick’s framework) cannot be performed here. To demonstrate newly acquired knowledge, either a pre-post test or a comparison to another educational modality would have been necessary. However, both are technically demanding and were not available for this investigation. Nevertheless, residents increased interest likely increased motivation, enjoyment, and inherent satisfaction, which may theoretically lead to a modification in behaviour (Level three of Kirkpatrick’s framework) and might promote intrinsic motivation. Second, our survey did not distinguish between different levels of previous experience (i.e., years of previous training) after graduation, or take into account differences in age and/or gender. However, it appears that knowledge (e.g., acquired as a medical student) may not necessarily be affected by the amount of time that has passed since graduation [60]. Also, a fundamental aspect of medical education appears to be the diversity of pre-existing requirements, with differences in situational conditions/settings especially relevant in complex environments [61]. Differences between educational settings, teacher-student interaction may affect medical educational research, which aims to bring forth clear, meaningful, and thus generalizable findings [62].

Third, and most importantly, we present observations and cannot conclusively identify the causes of relationships. Due to the shift work on the ICU, residents’ ability to participate was often limited. This may have influenced the results of the assessment. Furthermore, the sample size was limited. And importantly, we are unable to provide objective data on the effects of the teaching sessions and are unable to conclude that such teaching sessions directly resulted in improved ICU care. Nevertheless, our observations imply that teaching of physiology is feasible, and our data may support the design of interventional studies aiming to collect such objective data.

Fourth, in line with the limitations mentioned above, our statistical analysis can only be considered of supportive nature, and, in light of the limited number of participants, must be interpreted with caution.

## Conclusions

In an environment with time constraints such as the ICU, a 15-minute interactive peer-led flipped classroom teaching session was considered feasible and generally appeared useful for teaching critical care physiology to ICU residents. Self-reported questionnaire data indicated that teaching sessions sparked interest and increased motivation. This may theoretically induce a modification in professional behaviour and might have promoted self-directed learning. We therefore support use of the respective teaching format in the ICU. Whether such teaching sessions will result in improved ICU care remains to be elucidated in subsequent studies.
